# Differential behavioral aging trajectories according to body size, expected lifespan, and head shape in dogs

**DOI:** 10.1007/s11357-023-00945-9

**Published:** 2023-09-23

**Authors:** Borbála Turcsán, Enikő Kubinyi

**Affiliations:** 1 MTA-ELTE Lendület “Momentum” Companion Animal Research Group, Department of Ethology, Eötvös Loránd University, Budapest, Hungary; 2 Senior Family Dog Project, Department of Ethology, Eötvös Loránd University, Budapest, Hungary; 3 ELTE NAP Canine Brain Research Group, Budapest, Hungary

**Keywords:** Behavioral aging, Canine cognitive dysfunction, Lifespan, Body size, Healthspan

## Abstract

The twofold life expectancy difference between dog breeds predicts differential behavioral and cognitive aging patterns between short- and long-lived dogs. To investigate this prediction, we conducted a cross-sectional analysis using survey data from over 15,000 dogs. We examined the effect of expected lifespan and three related factors (body size, head shape, and purebred status) on the age trajectory of various behavioral characteristics and the prevalence of canine cognitive dysfunction (CCD). Our findings reveal that, although age-related decline in most behavioral characteristics began around 10.5 years of age, the proportion of dogs considered “old” by their owners began to increase uniformly around 6 years of age. From the investigated factors, only body size had a systematic, although not gradual, impact on the aging trajectories of all behavioral characteristics. Dogs weighing over 30 kg exhibited an earlier onset of decline by 2–3 years and a slower rate of decline compared to smaller dogs, probably as a byproduct of their faster age-related physical decline. Larger sized dogs also showed a lower prevalence of CCD risk in their oldest age group, whereas smaller-sized dogs, dolichocephalic breeds, and purebreds had a higher CCD risk prevalence. The identification of differential behavioral and cognitive aging trajectories across dog groups, and the observed associations between body size and the onset, rate, and degree of cognitive decline in dogs have significant translational implications for human aging research, providing valuable insights into the interplay between morphology, physiological ageing, and cognitive decline, and unravelling the trade-off between longevity and relative healthspan.

**Supplementary Information:**

The online version contains supplementary material available at 10.1007/s11357-023-00945-9.

## Introduction

Animal models have long been utilized to explore the complex mechanisms of aging. Among them, the domestic dog has rapidly become a prime model, particularly for studying behavioral and cognitive aging [1–3]. The protective human environment, combined with extensive veterinary care, has resulted in the doubling of dogs’ lifespan compared to wild wolves [1]. One of the reasons why the dog is an exceptional model for aging research, is that its longer lifespan allows for the observation of natural physiological, behavioral, and cognitive declines [1]. Moreover, the average life expectancy of dogs shows a more than two-fold difference between breeds [4], suggesting a potential relationship between the expected lifespan and cognitive aging. Despite this, relatively little is known about whether or how individual life expectancy is linked to the magnitude, timing, or speed of its age-related behavioral and cognitive decline.

This within-species variation in lifespan is closely related to variation in body size: on average, smaller individuals live longer than larger ones [4, 5]. Similar lifespan-size relationships have been described in many other species, including humans [6–9], rodents [10], and horses [11]. In the case of dogs, this inverse size-lifespan relationship is remarkable due to a significant 50-fold difference in body weight between the smallest and largest breeds, contributing to a median lifespan of 6.5 years for giant breeds such as great Danes (50–80 kg) and 14.6 years for lapdogs such as toy poodles (2–4 kg) [12].

The inverse relationship between size and longevity within a species runs counter to one of the most robust patterns in the biology of aging; that is, within a given taxon, larger species tend to live longer than smaller ones (see a review in [5]). To reconcile the conflicting trends observed within and between species, Rollo [10] proposed that the driving factor behind the differences in intraspecific longevity is not size but rather growth rate. Based on his meta-analysis of the reviewed mice and rat data, he concluded that larger individuals have higher growth rates, which can result in higher rates of oxidative cellular damage during early life, potentially having long-term negative effects on the animals’ health maintenance and longevity. With respect to dogs, however, this relationship does not seem to hold. In dogs, smaller breeds demonstrate faster growth rates compared with larger breeds, but larger breeds are in a growing phase for a longer period of time compared with small breeds [13–15]. As for how these differences in (early) growth patterns among sizes translate to differences in expected lifespan, there are plenty of theories and life-history traits suggested as candidates (see, for example, [13, 16, 17]), but consensus has not yet been reached. Even though the exact physiological mechanisms underlying the body size – lifespan trade-off is still unknown, mathematical models on mortality data from various breeds suggest that it has to do with aging rates. Kraus et al. [18] tested three alternative models on the mortality trajectories to find the pattern that best explains the body size-lifespan relationship. They found no difference in the baseline mortality across sizes, and also largely no difference in the onset of senescence (the age when mortality starts to increase), except for the somewhat earlier start of breeds over 50 kg. However, they found a strong positive relationship between size and the aging rate. Larger breeds showed a more rapid increase in mortality hazard after the onset of senescence than smaller breeds. Thus, they concluded that larger breeds have a shorter lifespan because, once they start aging, they age at a faster rate compared to smaller breeds.

However, there are two points to keep in mind. Firstly, body size is not the only factor contributing to the large variability in dog longevity. There are substantial differences in expected lifespan between breeds of similar size [4, 19], in part due to varying susceptibility to diseases [20–22]. A typical example of morphological susceptibility is the difference in expected lifespan based on head shape. Extreme brachycephalic (short-nosed) breeds have an expected lifespan that is 3–4 years shorter than meso- and dolichocephalic breeds of similar weight [23], presumably due to brachycephalic dogs’ higher predisposition to various diseases, including upper respiratory diseases (i.e., Brachycephalic Obstructive Airway Syndrome), allergies, and corneal ulceration [24–26]. In terms of genetic susceptibility, one example is the difference in average lifespan between purebred and mixed-breed dogs [19, 22, 27]. Mixed-breed dogs live considerably longer than purebreds within each size category, which can be attributed to hybrid vigor; that is, mixed-breed dogs have a much lower degree of inbreeding and are less likely to be homozygous for deleterious genes [28–30].

Secondly, an extended lifespan does not necessarily mean an extended healthspan as well. Aging is commonly accompanied by deteriorations in a variety of behavioral and cognitive functions, which can lead to the development of neurodegenerative disorders that significantly impact an individuals’ quality of life [1–3]. For example, aged dogs may develop pathological cognitive impairments, referred to as canine cognitive dysfunction syndrome (CCD) [31, 32], which not only show high phenotypic similarity to cognitive symptoms in aged humans but also share some physiological characteristics suggesting similar neuropathological pathways [33]. The decline in the physical and cognitive functions in older dogs poses challenges not only for the dog but also for its owner. Dog owners may prioritize higher quality over longer quantity when it comes to their dogs’ lives [22, 34, 35]. Consequently, a shorter expected lifespan in breeds may not necessarily pose a severe welfare problem if it is accompanied by a relatively long healthspan and a lower prevalence of CCD. Similarly, a longer life expectancy may not be advantageous if it only means spending a longer period in poor health. Only two studies have investigated the effects of size or expected lifespan on behavioral and cognitive aging to date. Salvin et al. [36] found numerous differences between size groups, longevity groups, and breeding groups (purebreds vs crossbreeds) in age-related changes in behavior and the prevalence of certain illnesses. However, they did not find a consistent direction in these differences, and thus could not support or reject the hypothesis that larger and/or short-lived dogs age earlier or have an increased rate of behavioral aging. Watowich et al. [37] found of the nine cognitive tests they investigated, the results of only two tests supported the hypothesis that breeds with shorter lifespans have a compressed cognitive age trajectory (i.e., age faster), while one test supported the ‘truncation hypothesis’ that longer- and shorter-lived breeds have the same cognitive age trajectory, but shorter-lived breeds experience limited cognitive decline. In the other six tests, they found no clear confirmation of either of the two contrasting hypotheses. Despite these results, Watowich et al. [37] still concluded that their findings were more in line with the truncation hypothesis than the compression hypothesis. Nevertheless, whether and how expected lifespan and its related risk factors are linked to the behavioral and cognitive aging trajectory is largely unexplored.

The overall aim of this study was to shed light on these associations using a large-scale international questionnaire. We aimed to describe and test the differential aging trajectories of behavioral changes, as well as the age at onset and age-related prevalence of CCD among dogs grouped by 1) expected lifespan, 2) body size, 3) head shape, and 4) purebred status (purebreds, and mixed-breed dogs). We specifically tested:

1. the *onset of decline,* which was represented by the breakpoint where the slope significantly changed;
2. the *rates of decline,* which described the steepness of slopes before and after the breakpoint.

**Fig. 1:**
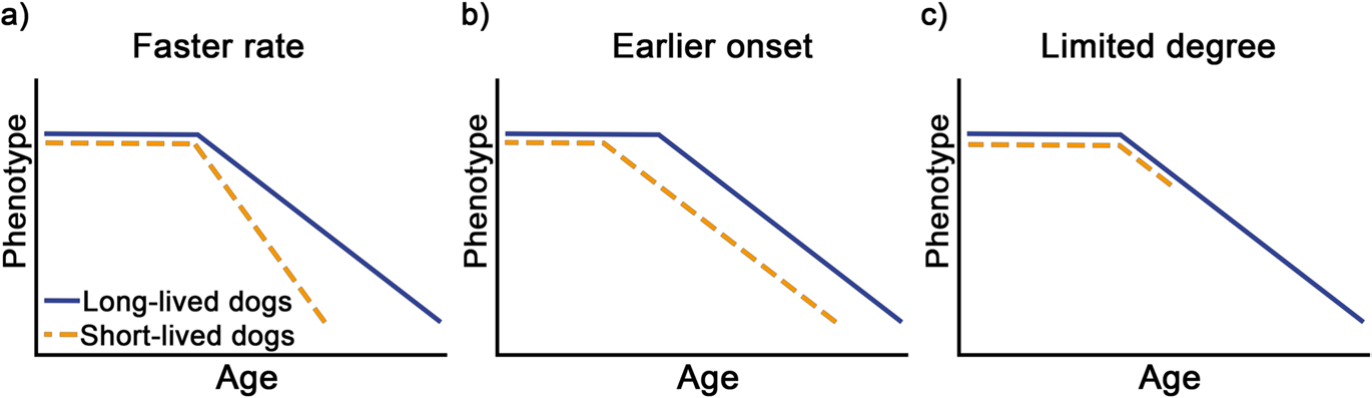
Outline of the three hypotheses raised in the current study. **a** The Faster rate hypothesis expects short-lived and larger breeds to have faster rate of decline and a shortened senior period. **b** The Earlier onset hypothesis assumes that the age-related decline starts at a younger age in short-lived and larger dogs. **c** The Limited change hypotheses expects short-lived and larger dogs to exhibit a limited degree of age-related decline as they die before more serious decline would start. Please note that contrary to their schematic representation here, these three hypotheses were not formulated to be mutually exclusive

Regarding expected lifespan and body size, we had three, not mutually exclusive hypotheses about how short-lived and large dogs age compared to long-lived and smaller dogs (Fig. 1):

(i) *Faster rate* (similar to the compression hypothesis in [37]): In short-lived and larger breeds, the rate of decline is faster, thus, the senior period is shortened. This pattern would be consistent with the results of previous studies [37], as well as with the faster aging rate described in [18].
(ii) *Earlier onset*: the onset of decline occurs at an earlier age in short-lived and larger dogs. This assumption is supported by the fact that larger dogs are considered ‘physiologically older’ than smaller dogs of the same chronological age in all age periods because their rate of growth already differs from birth to adulthood [36]. This would also align with the current veterinary opinion that larger dog breeds require “geriatric care” much sooner, between 6–9 years of age, compared to smaller breeds which require it between 9–13 years of age [38].
(iii) *Limited degree* (similar to the truncation hypothesis in [37]): short-lived and larger dogs exhibit a limited degree of age-related decline compared to their long-lived and smaller conspecifics as they die before more serious decline would start. This is supported by studies which have found that small and large breeds are differentially susceptible to certain diseases. Small breeds are more likely to die from what their owns consider “old age” [18] or from diseases associated with old age, such as neurological, urogenital, or endocrine diseases, while large breeds have an increased mortality risk from musculoskeletal and gastrointestinal diseases [4, 12, 39]. It is possible that the risk for diseases in the musculoskeletal and gastrointestinal systems show a faster rate of increase compared to the others, so at the time of death, the neural and endocrine systems, which may be more directly responsible for the behavioral and cognitive symptoms of aging, are still largely spared.

In the case of purebred status, there is some evidence for a higher absolute rate of aging (i.e., steeper slope of the mortality curve) in purebreds compared with mixed-breeds [30], suggesting the *Faster rate* hypothesis. However, purebred dogs are supposed to have a generally higher predisposition for (genetic) diseases, and that is why they live shorter than mixed breeds. Consequently, the *Limited degree* hypothesis is also plausible, that is, purebred dogs show a smaller degree of behavioral and cognitive decline compared to mixed-breeds because they experience more pronounced cognitive decline only for a limited time before they die of somatic causes.

Regarding head shape, although it is also a known risk factor for several diseases, only breeds with extreme brachycephalism have increased morbidity because of their health problems, thus it concerns only a few breeds [40]. On the other hand, the cephalic index (defined as the ratio between the width and length of the skull), even in continuous form, has been shown to affect the brain morphology [41], and the behavior [42] of the dogs, indicating that this factor may play a role in behavioral and cognitive aging, as well. Because of this, and because of the lack of information about this association in the literature, we included head shape in our analysis, mainly as an explorative measure. As such, we had no hypothesis for this factor, however, we generally expected brachycephalic dogs to be different from meso- and dolichocephalic dogs because of the few extremely brachycephalic breeds.

Finally, we were interested in determining at what age dogs are considered old by their owners. We assumed that it varies based on factors such as breed and size, as mentioned above. Based on the observations that age-related behavioral changes in dogs are rarely reported to veterinarians [32, 43], and most of the owners are rather disinclined to accept that their dogs started to show any behavioral changes indicative of aging [44], we expected that the ratio of “old” dogs would start to increase sometime after the behavioral aging had already begun.

## Method

### Ethics

The survey we used did not collect any sensitive or personal information about the owner. Participation in the study was voluntary and anonymous, and the owners were informed of the purpose of the study and that the collected data would be used in scientific analyses. The ethical permission number is PE/EA/2019–5/2017.

### Procedure

The survey we used consisted of three parts (Table 1). Part 1 contained items about the basic demographics and individual features of the dog, such as age, sex, neuter status, breed, and size. The last question in this part asked the owner if they think the dog is "old", i.e., if they see any signs of aging in the dog regardless of its actual age. Part 2, entitled "General behavior" consisted of 9 characteristics that were selected based on the CCD checklist of [31] and the results of a previous survey study as the characteristics that most strongly change with age [45]. Each characteristic was rated on a 1–10 scale, with only the extremes specified. Part 3, entitled "CCD symptoms", contained 5 questions selected from [31, 45, 46] to cover the main symptoms of the canine cognitive disorder syndrome. In these questions, the owners were asked to rate each symptom on a five-point frequency scale adapted from [46]. The owners were asked to consider the last 6 months when answering the questions in Parts 2 and 3. The survey was available in seven languages (English, German, French, Italian, Spanish, Portugal, and Hungarian), and was distributed online. The main platform was Facebook, where we targeted groups related to ethology, pets, dogs, breeds, dog behavior and dog training, and the participants were encouraged to share the post in other groups. Additionally, we also sent the link to the survey and a short article about the topic to several colleagues working abroad, as well as to journalists and bloggers who had covered dog- or ethology-related topics before.

**Table 1:** Items of the survey and descriptive statistics of the sample population (*N* = 16,818)

Question	Descriptive statistics
PART1 Demographics and individual features	
Age of the dog	range: 0.83—22.06 years, mean ± SD: 8.67 ± 4.10 years
Breed	purebred: 9,949 (59.2%) (286 breeds), mixed-breed: 6,727 (40.0%), not reported: 142 (0.8%)
Height at shoulders	range: 10 – 94 cm, mean ± SD: 43.7 ± 15.5 cm
Weight	range: 1.1 – 92 kg, mean ± SD: 19.6 ± 13.1 kg
Sex and reproductive status	intact male: 22.3%, intact female: 13.3%, neutered male: 25.7%, neutered female: 38.8%
In your opinion, is your dog old? (Do you see any signs of aging in your dog, independent of its actual age?)	no: 47.6%, yes: 52.4%
PART 2 General behavior, the items are to be rated on a 1–10 scale with the extremes specified	
Reactivity: how strongly or how fast your dog reacts to different stimuli (e.g., strange sound, new object, doorbell, etc.)	1: barely responsive, does not seem to be interested
	10: reacts immediately or strongly to almost everything
Activity: how much your dog likes to move around and participate in different types of activities?	1: quite lazy / apathetic, would rather just sleep the whole day
	10: highly energetic, always on the go
Learning ability: how quick is your dog to learn new tasks / actions?	1: very slow, seems to have difficulties with learning anything new
	10: learns very fast, needs only a little practice to get a new task
Motivation: how easy is it to motivate your dog to perform a task?	1: very hard to motivate him/her, not much interested in food or toys
	10: very easily motivated, would do anything for a treat or its favorite toy
Playfulness: how much your dog likes to play with toys or with others?	1: not interested in playing at all
	10: always eager and ready to play
Working performance: how reliable your dog performs tasks he/she has already learned?	1: not reliable, he/she does not seem to remember what he/she already learned
	10: once he/she learned something, he/she performs it reliably, no matter what
Social behavior towards owner: how much affection your dog shows towards you?	1: not much, does not seek petting or cuddling, does not really greet me when I arrive home
	10: very much, constantly seeks and enjoys being petted and cuddled
Social behavior towards strangers: how friendly your dog is towards strangers?	1: fearful or aggressive towards strangers, rather avoids them if he/she can
	10: very friendly to strangers, eager to approach them and seeks interaction
Social behavior towards other dogs: how friendly your dog is towards other dogs?	1: fearful or aggressive towards other dogs, rather avoids them if he/she can
	10: very friendly to other dogs, eager to approach them and seeks interaction
PART 3 CCD symptoms, the items are to be rated on a 1–5 scale: 1: never seen it happen; 2: happened at least once within the last 6 months; 3: happens at least once per month; 4: happens several times per month; 5: happens several times a week	
How often does it happen that your dog …	
…gets lost in familiar places	
…seems to be clumsy (knocks items accidentally, stumbles on the stairs, gets stuck somewhere (under the bed, in a corner))	
…gets frightened by familiar people, does not recognize them	
…changes in sleeping habits (restless during night, or sleeps more than usual during daytime)	
…elimination problems (has “accidents” in the house, or eliminates at uncommon locations)	

### Subjects and data preparation

We collected *N* = 17,428 responses from May 2018 to February 2022, from which *N* = 261 were duplicate entries. These duplicates were removed from the dataset. Dogs with no or unreliable age data reported (*N* = 116) were also excluded, as well as dogs under 10 months of age (*N* = 233), as they presumably had not yet reached their adult size. After these exclusions, *N* = 16,818 dogs remained in the full dataset. However, additional eligibility criteria for inclusion into the analysis were set for each factor (lifespan, body size, head shape, and purebred status) (see detailed below). There were 57 countries represented in the sample, but the majority (~ 80%) of the responses came from Hungary (36.5%), Brazil (25.4%), and Germany (17.9%). Further descriptive statistics of the sample are presented in Table 1.

#### Expected lifespan

The expected mean lifespan estimates for purebred dogs were obtained from Kraus et al. [4]. They obtained mortality data of over 40.000 dogs from a public database of the Finnish kennel club and estimated mean lifespan for over 100 breeds. This database presents a clear advantage regarding the quality of the data over alternative sources (e.g., [12, 22, 35]) as this study controlled for the potential bias in lifespan estimates due to right-censored age-at-death data and also a bias due to changing popularity and registration numbers. We were able to assign a lifespan estimate to 7,784 individuals representing 110 breeds (see breed-level details in SI 1). Since there are no officially accepted or even traditionally used thresholds in the literature for differentiating short-, medium-, and long-lived breeds, we divided the subjects into three approximately equal-sized subpopulations: short-lived (mean lifespan less than 11.4 years, *N* = 2,589), medium-lived (mean lifespan between 11.4 and 12.3 years, *N* = 2,653), and long-lived (mean lifespan greater than 12.3 years, *N* = 2,542) (Table S1 in SI 2).

#### Body size

The owners were asked to provide data about the height and weight of their dogs. However, it was deemed necessary to assess the reliability of this owner-reported size data before it could be used in further analyses to avoid incorrect guesses or weight issues affecting the results (similar to previous studies, e.g., [36, 45]). The reliability was determined differently for purebreds and mixed-breed dogs. In the case of *purebreds*, the owner-reported size data was compared to the weight and height range from the breed standards (FCI, AKC) or Wikipedia if no specification was given in the standard. The accepted range was defined as the minimum height/weight of females and the maximum for males, with a deviation of ± 5 cm or 1 kg allowed. If the owner-reported size data was within this range, we used that data for further analyses. If the reported size data was outside this range (*N* = 1,848 (18.5% of the purebred dogs) for height and *N* = 2,372 (23.8%) for weight) or no size data was reported (*N* = 1,971 (19.7%) for height, *N* = 273 (2.7%) for weight) we replaced it with the breed’s mean standard height or weight, calculated separately for males and females if the breed standard specified different range for the sexes.

The reliability of the owner-reported data for *mixed-breeds* and dogs of unknown breed was determined based on the height: weight ratio. First, we excluded mixed-breed dogs and dogs of unknown breeds for which either height or weight information was missing (*N* = 1,068). Next, we divided all the dogs into 40 weight groups based on a 1 kg range from 1 to 35 kg, and a 5 kg range from 35 to 60 kg, with the last group being for dogs weighing more than 60 kg (*N* = 72–622 purebreds per group). We defined the minimum and maximum height for each weight group based on the purebred dogs’ data. With more than 200 breeds in our sample, we expected all body shape types to be represented among the purebreds. This height range was used as a threshold for mixed and unknown breed dogs in the same weight group. If the height data provided by the owner was within this range, both height and weight data were considered reliable (*N* = 5,343, 91.7% of the cases). Otherwise, both size data were considered unreliable (*N* = 481, 8.3% of the cases).

The dogs’ categorization into size groups was based solely on their weight. Reliable weight data was available for *N* = 15,270 individuals. The dogs were divided into six size groups based on the classification provided by Salt et al. [47]. This classification categorized the dogs into six weight groups: toy (< 6.5 kg, *N* = 2,264); miniature (6.5- < 9 kg, *N* = 1,597); medium-small (9- < 15 kg, *N* = 2,900); medium-large (15- < 30 kg, *N* = 4,910); large (30–40 kg, *N* = 2,438); giant (> 40 kg, *N* = 1,161) (SI 1, Table S1 in SI 2). We decided to use this classification over the conventional size groups because it was determined by statistical means (cluster analysis) instead of a rule of thumb and was created based on the growth rate of the dogs, which is consistent with our hypotheses.

#### Head shape

The breed-average cephalic index estimates for purebred dogs were obtained from the merged dataset of Stone et al. [48] and our own dataset. We used the same data collection and cephalic index calculation methods as described in [48] on standardized photographs as detailed in [42]. We were able to assign a cephalic index (CI) estimates to 7,241 individuals representing 99 breeds (see breed-level details in SI 1). Since there are also no officially accepted cut-off values in the literature for defining specific head shape categories based on the cephalic index, we used the method as in the case of the lifespan groups and also as applied in [49] and divided the subjects into three approximately equal-sized subpopulations. Although we determined the cut-off values based on the N of dogs in each group, the resulting values also – accidentally – match those used in anatomy [41]: breeds with CI lower than 51 were classified as dolichocephalic (*N* = 2,339), breeds with CI ranging between 51 and 59 as mesocephalic (*N* = 2,437), and breeds with CI above 59 as brachycephalic (*N* = 2,465) (Table S1 in SI 2). Within this latter group, there were only 6 breeds (< 500 individuals in total) with extreme brachycephalic head shape (CI > 80).

#### Purebred status

The purebred status of the dog was categorized based on the owners’ answers to the “Breed of the dog” question. In this question, the owners were asked to indicate the breed of their dog if the dog was a purebred, or the ancestry of the dog if their dog is a mix and they knew the breed of the dog's parents, or the mixed-purebred status only if they had a mixed-breed dog and did not know the ancestry. The dog was categorized as purebred when the owner’s answer contained only a single breed, and as mixed-breed, if the owner listed more than one breed or answered mixed-breed. Purebred status information was available for *N* = 16,676 individuals, with *N* = 142 individuals not being involved in these analyses due to missing breed data. The purebred dogs (*N* = 9,949) included representatives from 288 breeds, with 26 breeds having more than 100 individuals, forming 37.0% of the full sample, and 156 breeds having less than 10 dogs, totaling 2.7% of the full sample (SI 1). The most popular breed, Labrador retriever, constituted 4% of the dataset.

#### Relationship between the grouping variables

As expected, the four grouping variables were not independent of each other (even if we ignore the fact that lifespan and head shape were analyzed only among purebreds), although there was no 1:1 correspondence between them. All pairwise comparisons can be found in Table S1 in SI 2.

### Statistical analyses

Our aim was to investigate the onset of and the rates of the age-related decline in various behavioral and cognitive traits and compare them across dog groups defined by the expected lifespan, body size, head shape, and purebred status. For these aims, we took the following steps:

#### Step 1 Defining the behavioral and cognitive characteristics

To assess the behavior of the individuals, we collected data regarding 9 behavioral characteristics, each rated on a 10-point scale, and 5 symptoms of the CCD, each rated on a 5-point scale). However, neither group of variables, especially the latter five, was expected to be independent from each other. Thus, we investigated the relationship between the different behaviors, and if a strong enough relationship was found, we obtained latent factors to reduce the number of redundant analyses. Both groups of behavioral variables were subjected to two exploratory factor analyses (EFAs), using the principal axis factoring method. The setup was the same for both analyses: we conducted EFAs with Varimax rotation, and the number of factors retained was decided using the Eigenvalue > 1 rule, as the number of items was relatively small in both analyses. Items which did not load > 0.4 on any components were removed in a stepwise manner. We used the Kaiser–Meyer–Olkin (KMO) measure and Bartlett Test of Sphericity to determine the sampling adequacy and Cronbach’s alpha coefficient to assess the internal consistency of the items for each factor.

Aside from the behavioral factors extracted from these analyses, we also investigated two binomial variables, namely the dog's high risk of suffering from CCD, and whether the owner considered the dog old. We defined a dog as having a high risk of CCD if they showed at least three of the five CCD symptoms at least once a month. The owners’ perception of their dog being old was categorized based on their answers to the question, “*In your opinion, is your dog old?*”. However, since the breakpoint and slope comparison analyses (see later) were not compatible with binary data, we transformed these variables into proportions. First, we divided the dogs into 19 age groups, each encompassing a one-year period, except for the last group, where, due to the low number of individuals, we merged all dogs into one group (19 + years old). Then, we calculated the proportion of dogs with a high risk of CCD and dogs considered “old” in each age group. When calculating these data separately for each dog group, we defined a threshold of 10 individuals as the minimum number of dogs in any given age group, where a proportion was calculated to avoid unreliable estimates. This criterion resulted in the exclusion of *N* = 19 dogs for the lifespan comparisons, *N* = 9 for body size comparisons, *N* = 19 for head shape comparisons, and *N* = 2 for purebred status comparisons. The oldest age group with enough data, separately for each dog group, is shown in Table S2 in SI 2.

#### Step 2 Investigating the age association of the above phenotypes

In the next step, we analyzed if the behavior characteristics and factors were related to age or not. If any of them were not significantly associated with age, there was no reason to analyze it further. Since many studies have now revealed that the relationship between age and behavior is not always linear (e.g., [50, 51]), we used regression with linear, quadratic, and cubic models to test the age trajectory of the variables. The different types of models would also indicate whether we should expect any (and how many) breakpoints on the aging curves, with linear, quadratic, and cubic models indicating zero, one, and two breakpoints in the curve, respectively. Phenotypes where no or very weak (R^2^ < 0.1) age association was found were not investigated in subsequent analyses.

#### Step 3 Identifying breakpoints on the aging curve (onset of decline)

Third, to identify the number and location of breakpoints on the age trajectory of the behavioral traits, we used the R package *Segmented v.1.6–0* [52]. First, we used the *'selgmented'* function which uses the Score test and the Davies test to select the number of breakpoints in a regression relationship via sequential hypothesis testing [53]. The Bonferroni correction was employed to account for multiple comparisons. If the function detected at least one significant or trend-level breakpoint, we used the ‘*segmented’* function to fit a segmented model onto the regression and obtain the location of the breakpoint. The ‘*confint’* function was used to compute the 95% confidence interval of the breakpoint and ‘*slope’* function was used to obtain the parameters of the slopes for each segment separately.

The breakpoint analysis was conducted both for the behavioral variables and for the proportion of “old” dogs, and both on the full sample (for descriptive purposes), and individually for each dog group we intended to compare (3 lifespan groups, 6 body size groups, 3 head shape groups, and 2 groups based on purebred status). As there was no targeted statistical test available to compare the breakpoint locations between groups, the comparison was based on the confidence interval (CI) of the breakpoints. A significant difference was considered if the 95% CI of two groups did not overlap.

#### Step 4 Comparing the slope of the curve among the dog groups (rate of decline)

Finally, to compare the rates of decline, i.e., the slope of the age trajectories between the dog groups, we used linear models. For each behavioral variable and for each grouping variable (lifespan, body size, head shape, and purebred status), two models were run, one before the breakpoint and one after it. To create the datasets for these analyses, the data for each group was divided at its respective breakpoint for the given behavioral trait. The data before the breakpoint for all groups was merged into one dataset, and the data after the breakpoint was merged into another. These datasets were analyzed separately using general or generalized linear models (GLM). The assumptions of the former model type were tested by visual inspection of the qq plot of the residuals and by assessing the homogeneity of variance using the Levene test. If these model assumptions were not met, generalized linear models were used with robust covariance matrix estimation. In both cases, the behavioral trait was entered as the dependent variable, the dog group as a fixed factor, and age as a covariate. The model included both main effects and the dog group x age interaction. If this interaction proved to be significant, it meant that the regression coefficients (i.e., slopes) of the age-behavioral trait equation were significantly different among the dog groups. In this case, we used pairwise comparisons as post-hoc tests to determine which groups differed from each other. To account for a large number of statistical comparisons, we used Bonferroni correction to adjust the threshold of significance separately for the models and for the post-hoc tests of each grouping variable. The effect sizes of the pairwise differences were estimated using partial eta squared in the cases of general linear models and odds ratio (Exp(B)) in the cases of generalized linear models. Finally, although the most popular breed takes up less than 5% of the total sample (SI 1), lifespan and head shape group comparisons are run on subsamples of purebred dogs, where the uneven representation of different breeds may bias the results. To investigate this possibility, we replicated the slope comparison analyses of these two groupings using generalized linear mixed models with breed entered as a random factor.

We used IBM SPSS (version 28.0) for the EFA and GLM analyses, all other analyses were run in R statistical environment (version 4.2.2) [54] using RStudio.

## Results

### Defining the behavioral and cognitive characteristics: results of the EFA

From the nine behavioral characteristics entered in the EFA, one (Sociability towards the owner) had to be excluded because of insufficient loadings (< 0.4). The remaining eight items formed two factors (KMO value > 0.8) that accounted for 65.83% of the total variance (Table 2). Sociability towards the owner characteristic had fallen out of the analysis because of low variance. The factors were labeled Liveliness-Trainability (six characteristics), and Sociability (two characteristics). Cronbach’s alpha values were > 0.6 for both factors, indicating adequate internal consistency.

**Table 2:** Rotated factor matrix of the EFA ran on the behavioral characteristics

Characteristic	Liveliness-Trainability	Sociability
Reactivity	**0.687**	0.004
Activity	**0.770**	0.153
Learning	**0.800**	0.128
Motivation	**0.751**	0.164
Playfulness	**0.718**	0.238
Working performance	**0.695**	0.138
Sociality towards strangers	0.086	**0.597**
Sociality towards dogs	0.137	**0.699**
Eigenvalue	3.952	1.314
Explained variance (%)	49.404	16.425
Cronbach's alpha	0.883	0.601

The Eigenvalues, explained variance, and Cronbach’s alpha values are presented at the end of the table. Loadings > 0.4 are in bold

Regarding the EFA run on the five CCD symptoms, all five remained in the analysis and formed one factor that accounted for 52.02% of the total variance. This factor was labelled The Severity of CCD symptoms (Table 3). Scores for both factors were extracted from the SPSS using the Regression method.

**Table 3:** Rotated factor matrix of the EFA run on the CCD symptoms

Characteristic	Severity of CCD symptoms
Gets lost in familiar places	0.672
Seems to be clumsy	0.749
Easily frightened by familiar people	0.516
Changes in sleeping habits	0.610
Elimination problems	0.611
Eigenvalue	2.601
Explained variance (%)	52.018
Cronbach's alpha	0.753

The Eigenvalues, explained variance, and Cronbach’s alpha values are presented at the end of the table

### The age association of the investigated phenotypes: results of age regressions

Liveliness-Trainability factor decreased moderately with age, and the relationship was stronger for the quadratic and cubic models than the linear model (Table 4). The same was true for four out of the six raw characteristics that make up this factor: Reactivity, Learning, Motivation, and Working performance. The remaining two raw characteristics (Activity and Playfulness) also decreased with age, but in their case, all three model types had similar strengths (Table S3 in SI 2).

**Table 4:** Relationship between the five behavioral traits and the age of the dogs

Behavioral trait	df2	Regression	R^2^	F	*P* value
Liveliness-trainability	16816	Linear	0.313	7665.947	< 0.001
	16815	Quadratic	0.342	4370.271	< 0.001
	16814	Cubic	0.342	2913.472	< 0.001
Sociability	16816	Linear	0.020	345.267	< 0.001
	16815	Quadratic	0.024	204.656	< 0.001
	16814	Cubic	0.028	162.735	< 0.001
Severity of CCD symptoms	16816	Linear	0.255	5762.692	< 0.001
	16815	Quadratic	0.319	3942.212	< 0.001
	16814	Cubic	0.319	2628.245	< 0.001
CCD-risk prevalence	17	Linear	0.827	81.365	< 0.001
	16	Quadratic	0.960	191.517	< 0.001
	15	Cubic	0.964	134.311	< 0.001
Proportion of “old” dogs	17	Linear	0.896	145.825	< 0.001
	16	Quadratic	0.941	127.418	< 0.001
	15	Cubic	0.984	313.254	< 0.001

The results of linear, quadratic, and cubic regressions are shown

The Sociability factor had a negligible association with age (highest R Square = 0.028), although all models were significant due to the large sample size. Similarly, neither of the three sociability-related raw characteristics changed markedly with age (R Square ranging from 0.005 to 0.056 (Table S3 in SI 2). So, neither the Sociability factor nor the three raw related characteristics were analyzed further.

The Severity of CCD symptoms factor, as well as the CCD-risk prevalence, increased moderately with age, and in both cases, the relationship was stronger for the quadratic and cubic models than the linear model. Finally, the proportion of “old” dogs also increased with age, and in this trait, the cubic model was stronger than both the linear and quadratic models (Table 4).

### Identifying breakpoints on the aging curve

The breakpoint analysis was first conducted on the full sample (*N* = 16,818), mainly for descriptive purposes. These analyses investigated the behavioral factors, the prevalence variables (Table 5), and the six raw characteristics that made up the Liveliness-Trainability factor (Fig. S1 in SI 2). These latter analyses were warranted because the results of the age associations suggested that the raw variables may follow different age trajectories, and if so, analyzing them as one factor could be misleading. After that, the breakpoints were analyzed separately for the different dog groups.

**Table 5:** Location (expressed in year-fraction) of the breakpoint(s) in the aging curves of various behavioral traits in different dog groups

Dog group	Liveliness-Trainability	Severity of CCD symptoms	CCD-risk prevalence	Proportion of “old” dogs
FULL sample	**10.41** (10.14–10.68)	**10.47** (10.28–10.66)	**10.31** (9.15–11.47)	**5.85** (5.14–6.56); **12.17** (11.66–12.68)
*Lifespan*				
Short-lived	**9.8** (9.1–10.6)	**8.6** (8.0–9.2)	**10.0** (8.4–11.6)	**6.8** (6.1–7.5); **10.6** (10.1–11.1)
Medium-lived	**9.5** (8.9–10.2)	**10.3** (9.9–10.8)	**11.1** (10.2–12.0)	**5.0** (4.0–6.1); **12.6** (11.7–13.4)
Long-lived	**10.7** (10.2–11.3)	**10.4** (9.9–10.8)	**10.7** (9.9–11.6)	**6.1** (5.6–6.6); **13.1** (12.6–13.5)
*Body size*				
Toy	**10.7** (10.1–11.4)	**10.8** (10.3–11.3)	**11.2** (10.2–12.3)	**5.9** (5.4–6.5); **12.7** (12.2–13.2)
Mini	**10.9** (10.1–11.7)	**10.0** (9.3–10.6)	**11.1** (10.0–12.1)	**6.2** (4.5–7.9); **13.9** (12.9–15.0)
Medium-small	**10.5** (9.8–11.1)	**10.5** (10.1–10.9)	**11.3** (10.4–12.1)	**6.1** (5.1–7.1); **13.0** (12.3–13.8)
Medium-large	**9.9** (9.4–10.3)	**10.5** (10.1–10.8)	**11.7** (10.7–12.7)	**5.8** (5.1–6.5); **12.1** (11.4–12.7)
Large	**9.1** (8.2–9.9)	**8.4** (7.8–9.9)	**9.7** (7.2–12.3)	**6.2** (4.7–7.6); **11.0** (10.0–12.0)
Giant	**7.1** (5.1–9.1)	**7.1** (5.9–8.3)	**7.9** (4.2–11.7)	**5.8** (4.6–7.0); **10.0** (9.1–10.9)
*Head shape*				
Brachycephalic	**10.7** (9.8–11.6)	**9.8** (9.1–10.5)	**10.9** (9.4–12.5)	**4.9** (3.6–6.2); **12.9** (12.0–13.9)
Mesocephalic	**10.4** (9.8–10.9)	**9.2** (8.7–9.7)	**10.9** (9.3–12.4)	**6.1** (5.2–6.9); **10.8** (10.0–11.6)
Dolichocephalic	**8.6** (8.0–9.3)	**10.9** (10.4–11.4)	**12.2** (11.4–12.9)	**7.9** (7.1–8.7); **10.8** (10.2–11.3)
*Purebred status*				
Purebred	**10.4** (10.0–10.7)	**10.5** (10.2–10.7)	**11.2** (10.5–11.9)	**5.7** (4.9–6.5); **12.0** (11.4–12.6)
Mixed-breed	**10.5** (10.1–11.0)	**10.6** (10.3–10.9)	**11.3** (10.7–11.8)	**5.9** (5.2–6.6); **12.9** (12.4–13.3)

The location of the breakpoints are presented in bold. Numbers in the brackets present the 95% of confidence interval. In the case of the Proportion of “old” dogs variable, two breakpoints were found in most dog groups, the one occurring at younger age indicating when the proportion starts to increase and the one occurring at older age when the proportion reaches a plateau

The analyses found a single significant breakpoint in the Liveliness-Trainability factor for both the full sample and all dog groups. Similarly, one breakpoint was found for six raw characteristics that made up the Liveliness-Trainability factor, and their location (ranging from 10.32 to 10.92 years, Fig. S1 in SI 2) were all in close proximity to each other and to the breakpoint of the Liveliness-Trainability factor itself (10.41 years). This justifies analyzing them as one factor.

In the Severity of CCD symptoms factor, the analyses found only one significant breakpoint in the full sample (at 10.47 years), but two significant breakpoints for half of the dog groups (the short-lived and medium-lived lifespan groups, the medium-small and medium-large size groups, the dolichocephalic head shape group, and both the purebred and mixed-breed groups). The secondly identified breakpoint occurred at a younger age than the firstly identified breakpoint; however, its significance was much weaker than the first, and it only differentiated a small increase in the factor scores before the more pronounced change in slope, which indicated the real start of aging. Since the breakpoint analysis was intended to analyze the onset of the age-related changes (which was captured by the firstly identified breakpoint) and because of practical reasons (i.e., the same number of breakpoints in all groups within a grouping variable is needed for slope comparison), only one breakpoint was extracted for all groups.

Regarding CCD-risk prevalence, one breakpoint was found in all analyses, although the breakpoint of the giant size group was only at the trend level (*p* = 0.068). On the full sample, the percent of dogs with CCD risk was around 7% when it started to increase significantly (see also in Table S4 in SI 2), and the timing (10.31 years) was close to the breakpoints of the two behavior factors.

Finally, in the case of the proportion of “old” dogs, the analyses found two breakpoints for the full sample and for all groups, although the secondly identified breakpoint was only at a trend level for the large and giant size groups (*p* = 0.068 and *p* = 0.075, respectively) and for all three head shape groups (*p* = 0.098–0.054). Just as in the case of CCD prevalence, the secondly identified breakpoint occurred at a younger age, but in this variable, this secondly identified breakpoint indicated the age when the proportion of “old” dogs started to steeply increase in the population. It occurred at 5.8 years of age on the full sample, and the ratio of dogs considered old by their owners was approximately 20% in the population at that time. The firstly identified breakpoint which occurred at an older age, indicated the age when the proportion of “old” dogs reached a plateau (i.e., the increase significantly slowed down). This happened at 12.2 years of age on the full sample, when the ratio of “old” dogs was around 90% in the population (Table S4 in SI 2).

**Fig. 2:**
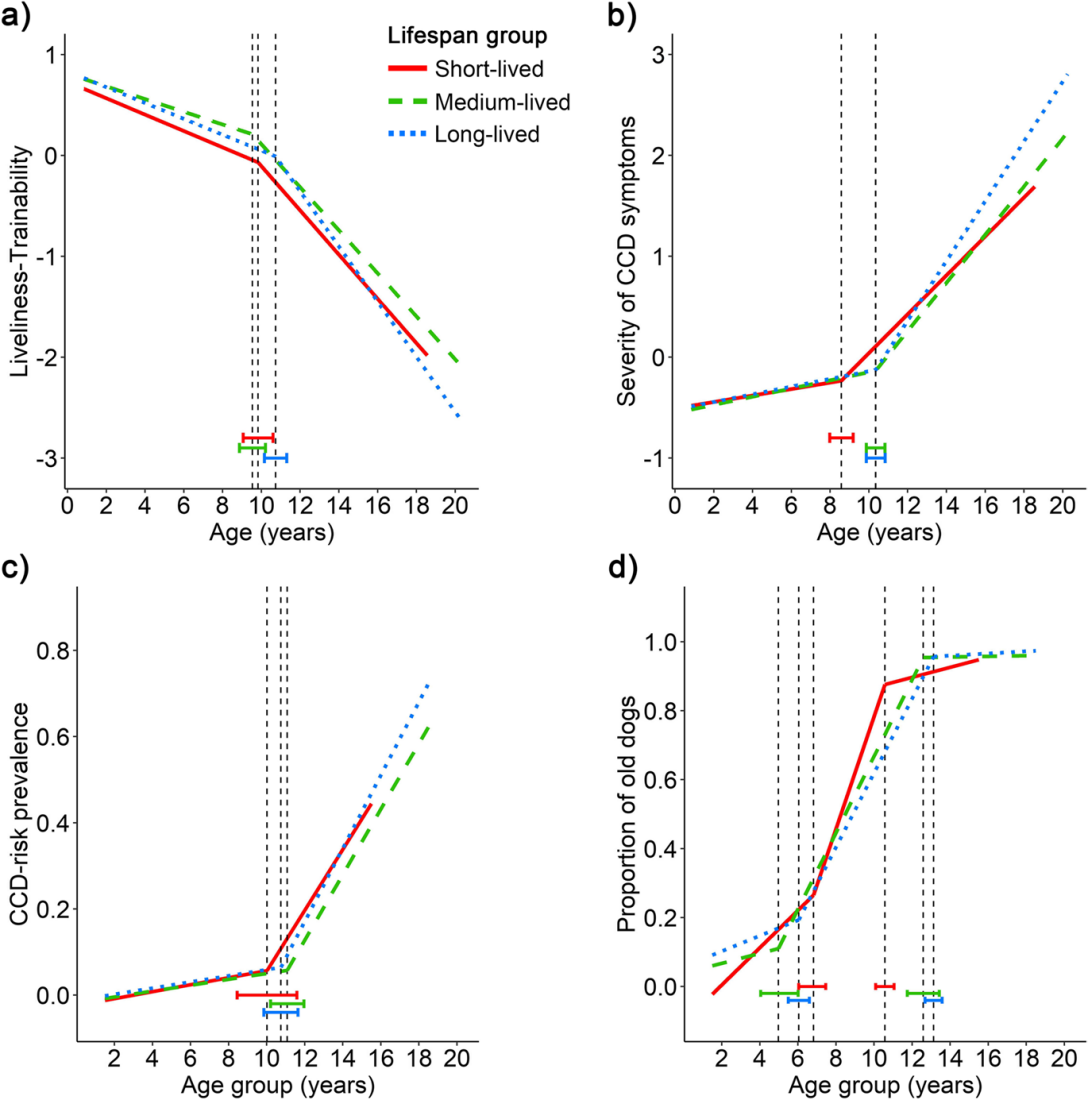
Aging trajectories of the four investigated variables: **a** Liveliness-Trainability, **b** Severity of CCD symptoms, **c** CCD-risk prevalence, and **d** Proportion of “old” dogs in the three lifespan groups: short-lived (mean lifespan < 11.4 years, *N* = 2,589); medium-lived (mean lifespan 11.4–12.3 years, *N* = 2,653); long-lived (mean lifespan > 12.3 years, *N* = 2,542). The vertical dashed lines indicate the location of the breakpoints (i.e., the age when the slope of the age trajectory changes significantly), the bars represent their 95% confidence intervals

When comparing the locations of the breakpoints among the lifespan groups (Fig. 2), there were differences in the Severity of CCD symptoms and proportion of “old” dogs (second breakpoint). In both variables, the breakpoints for short-lived dogs occurred earlier than medium- and long-lived dogs, while no significant differences were found between the medium-, and long-lived lifespan groups.

**Fig. 3:**
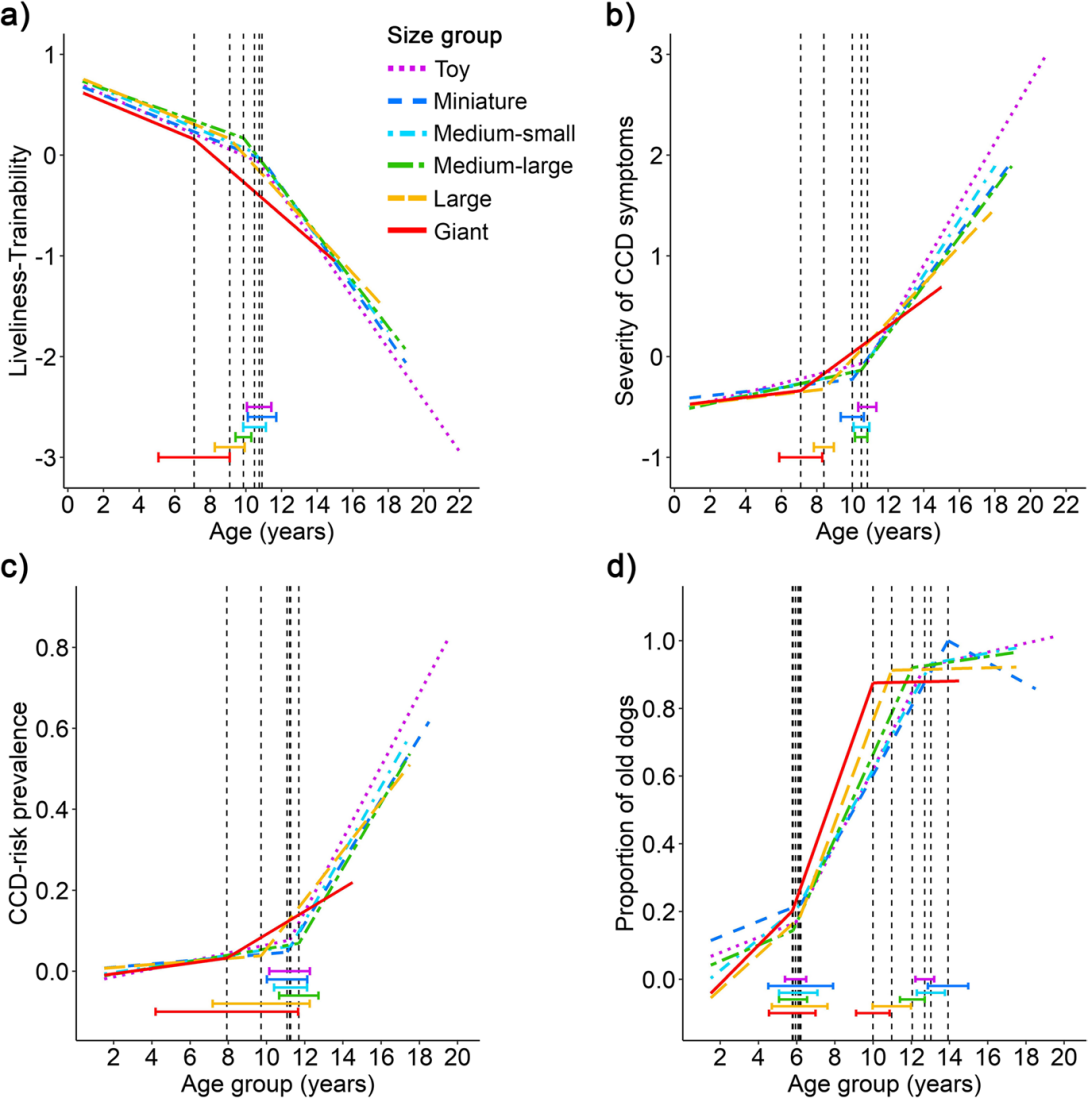
Aging trajectories of the four investigated variables: **a** Liveliness-Trainability, **b** Severity of CCD symptoms, **c** CCD-risk prevalence, and **d** Proportion of “old” dogs in the six body size groups: toy (< 6.5 kg, *N* = 2,264); miniature (6.5- < 9 kg, *N* = 1,597); medium-small (9- < 15 kg, *N* = 2,900); medium-large (15- < 30 kg, *N* = 4,910); large (30–40 kg, *N* = 2,438); giant (> 40 kg, *N* = 1,161). The vertical dashed lines indicate the location of the breakpoints (i.e., the age when the slope of the age trajectory changes significantly), the bars represent their 95% confidence intervals

The location of the breakpoints in the aging curves differed among the different body size groups for the Liveliness-Trainability and Severity of CCD symptoms factors, as well as the second breakpoint in the proportion of "old" dogs (Fig. 3). In all three variables, giant and large sized dogs were different from the smaller groups, while no significant differences were found among the four smaller groups. Specifically, giant dogs had earlier breakpoints than all other groups except the large ones in all three variables. The large dogs also had an earlier breakpoint than the toy and miniature dogs in the Liveliness-Trainability factor, earlier than all other groups except the giant in the Severity of CCD symptoms factor, and earlier than the toy, miniature, and medium-small dogs in the proportion of “old” dogs variable (second breakpoint).

**Fig. 4:**
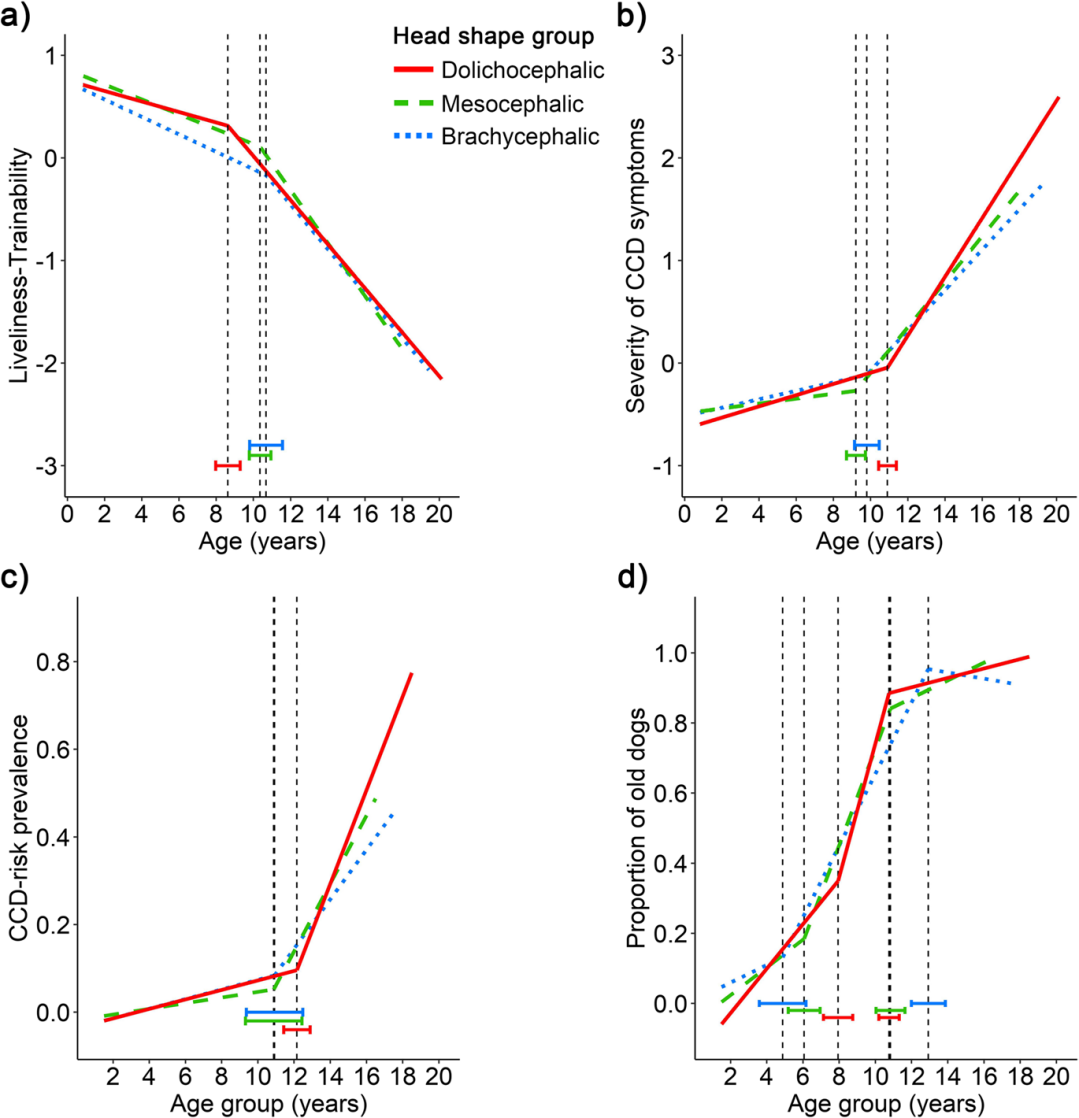
Aging trajectories of the four investigated variables: **a** Liveliness-Trainability, **b** Severity of CCD symptoms, **c** CCD-risk prevalence, and **d** Proportion of “old” dogs in the three head shape groups: brachycephalic (cephalic index > 59, *N* = 2,465); mesocephalic (cephalic index 51–59, *N* = 2,437); dolichocephalic (cephalic index < 51, *N* = 2,339). The vertical dashed lines indicate the location of the breakpoints (i.e., the age when the slope of the age trajectory changes significantly), the bars represent their 95% confidence intervals

In the case of head shape groups (Fig. 4), the breakpoint for dolichocephalic dogs was found to be at a younger age than that of meso- and brachycephalic dogs in the Liveliness-Trainability factor. However, in the Severity of CCD symptoms factor, the breakpoint for dolichocephalic dogs was at an older age. Dolichocephalic dogs also had the first breakpoint of the proportion of “old” dogs variable an older age, while brachycephalic dogs had the second breakpoint in this variable at an older age than the other two groups.

**Fig. 5:**
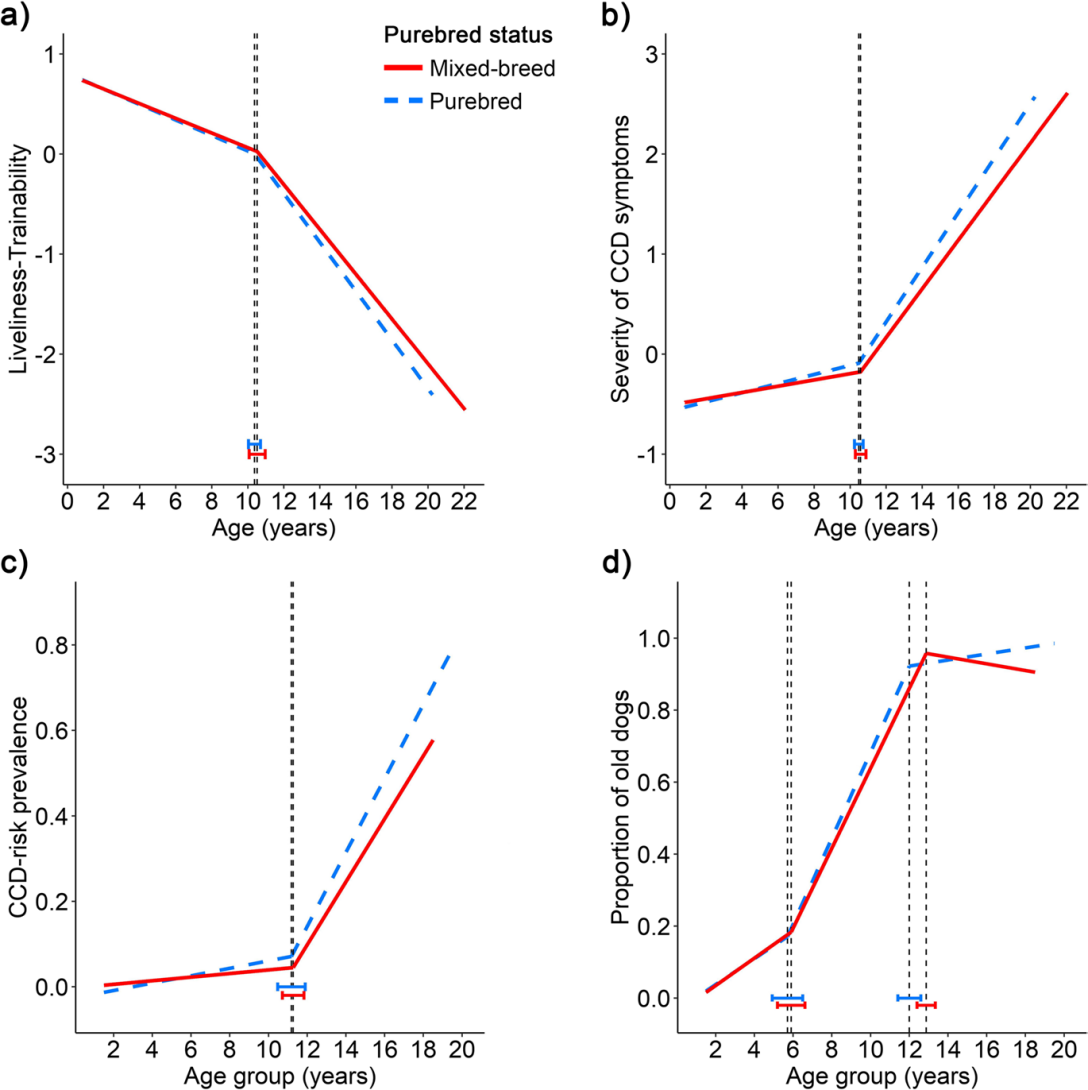
Aging trajectories of the four investigated variables: **a** Liveliness-Trainability, **b** Severity of CCD symptoms, **c** CCD-risk prevalence, and **d** Proportion of “old” dogs in purebred (*N* = 9,949) and mixed-breed dogs (*N* = 6,727). The vertical dashed lines indicate the location of the breakpoints (i.e., the age when the slope of the age trajectory changes significantly), the bars represent their 95% confidence intervals

Finally, there was no difference in the breakpoints between purebred and mixed-breed dogs (Fig. 5).

### Comparing the rate of decline among dog groups

The differences in the slopes of the aging curves were investigated by analyzing the dog group x age interaction in general or generalized linear models. Model details, including sample sizes, parameter estimates, and effect sizes are provided in SI 3. A simplified overview of the results is presented in Table 6.

**Table 6:** Overview of the main results

	Lifespan	Body size	Head shape	Purebred status
*Liveliness-trainability*				
Breakpoint	no difference	giant < all others; large < toy, miniature	dolichocephalic < meso- and brachycephalic	no difference
Slope before	no difference	no difference	dolichocephalic < brachycephalic	no difference
Slope after	short- and medium-lived < long-lived	giant < all others except large; large < toy	no difference	no difference
*Severity of CCD symptoms*				
Breakpoint	short-lived < medium- and long-lived	giant, large < all others	dolichocephalic > meso- and brachycephalic	no difference
Slope before	no difference	no difference	dolicho- and brachycephalic > mesocephalic	mixed-breed < purebred
Slope after	short- and medium-lived < long-lived	giant < all others; large > toy, medium-small	dolichocephalic > meso- and brachycephalic	no difference
*CCD-risk prevalence*				
Breakpoint	no difference	no difference	no difference	no difference
Slope before	no difference	toy > miniature	no difference	no difference
Slope after	no difference	giant < all others except large	dolichocephalic > brachycephalic	no difference
Prevalence in the oldest age group	no difference	giant < all others < toy	dolichocephalic > meso- and brachycephalic	mixed-breed < purebred
*Proportion of “old” dogs*				
Breakpoint #1	no difference	no difference	dolichocephalic > meso- and brachycephalic	no difference
Breakpoint #2	short-lived < medium- and long-lived	giant, large < toy, miniature, medium-small; giant < medium-large < miniature	brachycephalic > meso- and dolichocephalic	no difference

In the case of breakpoints, the " < ” and " > " signs indicate if the breakpoint (the starting point of the change) was at a significantly younger ( <) or older ( >) age. In the case of slopes, these signs indicate the steepness of the trajectories, with less steep ( <) trajectory meaning slower, more gradual change (i.e., smaller absolute value of the slope), and steeper ( >) trajectory a faster change

#### Lifespan groups

When comparing the lifespan groups, differences were found between the short-lived and the other two groups (Fig. 2). In Liveliness-Trainability factor, there was no difference in the slope before the breakpoint (dog group x age interaction, *p* = 0.102), but the interaction was significant after the breakpoint (F = 5. 997, *p* = 0.003), with short- and medium-lived dogs having a less steep trajectory than long-lived dogs.

A similar pattern was found in the Severity of CCD symptoms factor with no difference before the breakpoint (*p* = 0.543), but a significant difference after it (Wald χ^2^ = 21.368, *p* < 0.001), and, again, short-lived dogs had a less steep trajectory compared to the other groups.

To account for the large variability in the different breeds’ frequencies in the sample, we replicated these four analyses using a mixed-model approach. These models showed the same results pattern, i.e., no difference before the breakpoint but significant differences after it, with long-lived dogs being different from the other two groups (see details in SI 3).

For the CCD-risk prevalence, no significant difference was found in the slopes either before the breakpoint (*p* = 0.917) or after (*p* = 0.555).

#### Body size groups

Regarding the body size groups (Fig. 3), no differences were observed in the slope before the breakpoint in the Liveliness-Trainability factor (*p* = 0.466). However, differences were found after the breakpoint (Wald χ^2^ = 26.613, *p* < 0.001), with giant dogs having a less steep trajectory than all smaller groups except for large dogs and large dogs having a less steep trajectory than toy dogs.

For the Severity of CCD symptoms factor, a significant slope difference was found both before the breakpoint (Wald χ^2^ = 17.589, *p* = 0.004), and after the breakpoint (Wald χ^2^ = 63.818, *p* < 0.001). Before the breakpoint, no pairwise difference was significant after correction for multiple comparisons using the Bonferroni method. After the breakpoint, giant dogs were found to have a less steep trajectory compared to all smaller groups except for large dogs, and large dogs had a less steep trajectory compared to toy and medium-small dogs.

Regarding CCD-risk prevalence, the slope differed before and after the breakpoint (F = 4.763, *p* = 0.001; F = 6.351, *p* < 0.001, respectively). Before the breakpoint, the toy group was found to have a steeper trajectory compared to the miniature group; while after the breakpoint, giant dogs had a less steep trajectory compared to all other groups except large dogs.

#### Head shape groups

Regarding the head shape groups (Fig. 4), we found a significant difference in the slope before the breakpoint in the Liveliness-Trainability factor (Wald χ^2^ = 12.032, *p* = 0.002). Dolichocephalic dogs had a less steep trajectory than brachycephalic dogs. No difference in slope was found after the breakpoint (*p* = 0.120).

In the Severity of CCD symptoms factor, before the breakpoint (Wald χ^2^ = 17.988, *p* < 0.001), both dolichocephalic and brachycephalic dogs had a steeper slope than the mesocephalic group. After the breakpoint (Wald χ^2^ = 9.234, *p* = 0.010), dolichocephalic dogs had a steeper trajectory than both other groups.

Like in the lifespan grouping, we also replicated these four analyses using a mixed-model approach to investigate if heavily represented breeds might drive some of these results. Again, the mixed models showed the same results pattern as the original ones (see details in SI 3).

In CCD-risk prevalence, the slopes differed only after the breakpoint (before: *p* = 0.284; after: F = 5.833, *p* = 0.013). In the latter case, dolichocephalic dogs had a steeper trajectory than brachycephalic dogs.

#### Purebred and mixed-breed groups

Lastly, there was no difference in the slopes of the Liveliness-Trainability factor between purebred and mixed-breed dogs either before or after the breakpoint (*p* = 0.406, *p* = 0.104, respectively) (Fig. 5).

In the Severity of CCD symptoms factor, before the breakpoint, the slopes differed between the groups, with purebred dogs having steeper trajectory than mixed-breed dogs (Wald χ^2^ = 12.921, *p* < 0.001). After the breakpoint, the difference in the slope did not reach the significance level (Wald χ^2^ = 3.369, *p* = 0.066).

Similarly, no difference was found in the CCD-risk prevalence either before (*p* = 0.101) or after the breakpoint (*p* = 0.141).

## Discussion

Our study has shown that expected lifespan, body size, and head shape are all associated with the patterns of behavioral and cognitive aging, but only body size had a systematic impact on the age trajectories of all investigated behavioral variables. Dogs weighting over 30 kg exhibited an earlier onset but a slower rate of decline in all investigated behavioral variables, resulting in a limited degree of age-related change compared to smaller size groups. We obtained more controversial results regarding the expected lifespan and head shape groups. Short-lived dogs had an earlier onset and a slower rate of decline but only in certain variables, while dolichocephalic dogs had earlier and later onset, as well as slower and faster rate of age-related change, depending on the behavioral variable investigated. Purebred status was not associated with behavioral aging, but purebred dogs had a higher risk to develop CCD compared to mixed-breed dogs. The second major finding was that the ratio of dogs that owners considered old began to increase in the population at a significantly younger age than the onset of any age-related behavioral changes. The starting point was largely the same (~ 6 years of age) for all dog groups.

### Differences across groups based on expected body size, head shape, purebred status, and expected lifespan

The main objective of this study was to explore the relationship between the phenotypic manifestation of aging and several variables (body size, head shape, purebred status, and expected lifespan).

#### Body size

In the case of the size groups, we found support for both the *earlier onset* and the *limited degree* hypotheses but also systematic proof against the *faster rate* hypothesis.

Giant (and, to some extent, also large) dogs started to change at a 1–3 years younger age compared to other groups in both Liveliness-Trainability and Severity of CCD symptoms factors, supporting the *earlier onset* hypothesis. These findings partly align with the results of Kraus et al. [18], who reported that the onset of senescence occurred earlier in breeds over 50 kg.

Giant (and, to some extent, also large) dogs also had a less steep slope after the breakpoint in Liveliness-Trainability, Severity of CCD symptoms, and CCD-risk prevalence variables, and these results directly contradict the *faster rate* hypothesis.

Finally, we also showed that in the oldest age group, the prevalence of CCD-risk decreased as body size increased. The two largest drops were observed between the toy (84.6%) and miniature (61.4%) groups, and between the large (40.0%) and giant (18.7%) groups. These results support the *limited degree* hypothesis (i.e., larger dogs experience a limited magnitude of age-related decline due to their shorter lifespan), and they are also in accordance with [55], who found that lower weight was associated with higher risk of CCD.

It is also worth mentioning that since we controlled for potential weight issues when assigning a weight category to the dogs, we expect that abnormal body conditions to have a negligible effect on the results.

Altogether, our results indicate that the behavioral and cognitive aging trajectories do not mirror the physiological aging patterns, considering that [18] reported a faster rate of aging in larger compared to smaller breeds based on mortality data. A possible explanation for this might be that the age-related behavioral changes we observed in the cases of the dogs weighting over 30 kg were not (solely) the direct results of brain aging, but rather the indirect consequences of age-related physical deterioration, accumulating illnesses, and degradation in sensory functions. For example, musculoskeletal problems like arthritis can directly reduce the activity level of the animal through pain and discomfort, and indirectly through owner-implemented changes in lifestyle and care practices (e.g., shorter walks, less play, less training exercise, and fewer opportunities to engage in intraspecific interactions, etc., [27]). These changes and the resulting lower levels of physical and mental stimulation are what can lead to behavioral changes reminiscent of the effect of cognitive aging [45, 56–58]. However, since these behavioral changes are the indirect result of physical health problems and not the direct result of neural (cognitive) deteriorations, their onset occurs at an earlier age, and their rate of change is slower. In other words, dogs weighing over 30 kg may be the most strongly affected by an early-onset physical decline (as suggested in [18]), and this physical decline and accumulating health problems are the main cause of their age-related behavioral changes, which occur before significant cognitive (neural) decline begins. This hypothesis is indirectly supported by the result of Chen et al. [59] that larger dogs showed faster age-related decline in health-related quality of life scores (especially in activity and comfort) than smaller dogs. Nevertheless, further studies are needed to find direct support for it.

Alternatively, the heterogeneity within the larger dog population may play a role in the slower rate of decline we observed. Lower-quality (less healthy) individuals may experience higher mortality rates, leaving behind a selected group of more robust individuals. This scenario could potentially lead to an attenuated decline in the larger dog group over time. Since our study did not directly investigate either of these hypotheses, we acknowledge the need for further exploration to elucidate the underlying mechanisms contributing to the observed patterns of decline in different size categories.

#### Head shape

With regards to head shape, we had no expectation about which aging characteristic should differ and in which direction across the groups, however, we generally anticipated that shorter-headed dogs would differ from the other groups. Contrary to this expectation, we found that mostly the long-headed group differed from the other two. Dolichocephalic dogs exhibited 2-year earlier decline in Liveliness-Trainability than brachy- and mesocephalic dogs. Their rate of change was slower before the breakpoint compared to brachycephalic breeds, and no difference was found in the after-break slope. The pattern was reversed in the Severity of CCD symptoms factor, where dolichocephalic breeds exhibited a 1-year later onset and more rapid decline both before and after the breakpoint compared to other groups. In the CCD-risk prevalence variable, dolichocephalic dogs demonstrated a faster decline after the breakpoint than the other two groups. In the oldest age group, CCD-risk prevalence was nearly twice as high (77%) in dolichocephalic dogs compared to the prevalence in the brachycephalic and mesocephalic groups (40% for both). Although these results contradicted our expectations, again, heterogeneity within subgroups, particularly among brachycephalic breeds, could influence the observed differences in the aging trajectories. We expected differences between the brachycephalic and other groups due to the former group including breeds with higher susceptibility to diseases However, if dogs from less healthy brachycephalic breeds (e.g., Pugs, English and French bulldogs) experience higher mortality rates at younger ages, it is plausible that the surviving brachycephalic individuals in the sample may disproportionately represent healthier breeds. This may explain why we found no difference between the brachycephalic and the mesocephalic groups.

Overall, our findings imply that dolichocephalic breeds may be more susceptible to CCD than other head shape groups, perhaps due to brain structure differences [41]. CCD is characterized by cortical atrophy and widening of the ventricles, and dolichocephalic dogs have a higher cortical-to-ventricular volume ratio [60]. Although additional research is needed to determine how and to what extent variations in the cortex/ventricle ratio correlate with CCD pathology, these findings could be significant for owners of dolichocephalic dogs when they consider their pets’ quality of life in their later years.

#### Purebred status

We had anticipated that purebred dogs would be less affected by age-related diseases such as CCD since they have shorter lifespans than mixed-breeds (*limited degree* hypothesis). Moreover, we also expected purebred dogs to have a faster rate of aging, parallel to their faster demographic aging rate reported in [30]. However, we found no support for the former and only a small support for the latter hypothesis. In the oldest age group, the CCD-risk prevalence was 81.8% among purebreds and only 54.8% among mixed-breeds, and mixed-breed dogs exhibited a slower rate of decline before the breakpoint in the Severity of CCD symptoms factor compared to purebred dogs. Consequently, it seems that the hybrid vigor of mixed-breeds helps them maintain their cognitive health for longer periods [29]. On the other hand, it is also worth noting that we found no significant difference between these groups in the onset of aging, nor in the age trajectories of Liveliness-Trainability and CCD-risk prevalence variables, suggesting that purebred status has a negligible association with behavioral and cognitive aging.

#### Expected lifespan

Since body size is the strongest predictor of the expected lifespan, we assumed that the performance of short-lived dogs would be similar to that of giant-sized dogs. The results partly supported this hypothesis with regards to the Liveliness-Trainability and Severity of CCD symptoms factors. Just as the giant size group, the short-lived dogs had an earlier onset of decline in the Severity of CCD symptoms and short- and medium-lived a slower rate of decline after the breakpoint in both factors. However, there was no difference in the CCD-risk prevalence trajectory between the lifespan groups, and no marked difference in the prevalence of CCD-risk among the oldest dogs (short-lived: 47.6%, medium-lived: 52. 6%, long-lived: 63. 6%, Table S2 in SI 2). This result is consistent with the findings of [32] but contrasts the results of the giant dogs. This discrepancy suggests that the advantage of having a lower CCD-risk prevalence is linked to giant body size and not to a short lifespan in general. Moreover, the lifespan groups contained only purebred dogs, while the size groups contained mixed-breeds, too, which could also partly explain the lower CCD-risk prevalence in the giant group.

### When do dogs start to age?

The second goal of this study was to investigate when dogs start to age, both from behavioral perspective and from the owners’ perspective. Specifically, we were interested in the age at which a dog is considered "old" by its owners, how it is related to the onset of behavioral aging, and if it differed across the dog groups.

Among the investigated behaviors, Sociability did not change markedly with age, in accordance with [51, 61], but contrary to [62, 63]. Most of the other remaining characteristics and factors followed a quadratic curve, indicating a significant change in the steepness of the slope, which we defined as the onset of age-related behavioral decline.

We found that the breakpoints in Liveliness-Trainability and Severity of CCD symptoms factors occurred close to each other (10.41 years and 10.47 years, respectively) in the full sample, and, in some cases, even at the exact same age within the different dog groups. The breakpoint analysis of the six raw behavioral characteristics largely agreed with the breakpoint of the Liveliness-Trainability factor (ranging from 10.32 to 10.92). Although the lack of variability among these characteristics contradicts previous findings, which suggest trait-dependent age-trajectories [51, 64], it also indicates that the raw variables which made up the factor did not follow markedly different trajectories, at least regarding the timing of age-related changes. These results together suggest that the age at which these breakpoints occurred marks the beginning of behavioral aging and the onset of age-related behavioral changes. The breakpoint in CCD-risk prevalence on the full sample also occurred around the same age as in all other traits (10.31 years), further confirming this age as the starting point of the behavioral decline and supporting the results of [65], which showed that severity of CCD symptoms begins to increase after the age of 10 years. However, contrary to the full sample, the breakpoint in CCD-risk prevalence within the different dog groups occurred approximately 1 year later than the breakpoint of the behavior factors, which could be due to the relatively rigorous way we defined having CCD-risk (i.e., showing at least 3 symptoms, each at least once in a month).

Contrary to the behavioral variables, the age trajectory of the proportion of dogs considered “old” followed a cubic curve, indicating the existence of two breakpoints. The first breakpoint indicated the age when the ratio of “old” dogs started to increase in the population. This breakpoint occurred at approximately 6 years, regardless of the expected lifespan, body size, and purebred status, at a significantly younger age than the onset of any age-related behavioral changes. Although there could be unmeasured behavior indicators where the dynamics of the age trajectory align better with the owner's perception, we believe that subtle changes in appearance (such as greying), sensory function, or health could accumulate around this age. The potential effect of the facial features on the owner perception is also suggested by the fact that head shape groups differed in the timing of the first breakpoint, it occurred around 2 years later in dolichocephalic dogs than in the other two groups. The second breakpoint indicated the age when the ratio of “old” dogs reached the plateau. Here, we found some differences among the dog groups, namely that giant, large, and short-lived dogs reached this point at a younger age, while brachycephalic dogs at an older age than the other groups.

### Limitations

First, we need to mention the cross-sectional nature of the study, which makes the results more easily biased by confounding factors, like the cohort effect (i.e., systematic differences between age groups (cohorts) due to shared experiences of the individuals born during the same time period).

Second, the data were obtained from volunteer owner reports and may be affected by sampling bias. The owners of dogs that age successfully may be more likely to participate in dog-aging studies, thus over-representing very old dogs in the sample. Therefore, the life expectancy differences among the groups were only partially reflected in the sample. If we consider the oldest age group in our sample, which contained at least ten dogs, we did not have enough giant dogs older than 15 years. On the other hand, there was not much difference in the number of geriatric dogs among the other sizes and groups, nor among the lifespan, head shape, and purebred status groups. The imbalance in the sample composition may partially explain why, among the size groups, mostly giant dogs differed from the others.

Third, some dog groups, including giant dogs, were underrepresented in the sample. This may reflect their rarity in the global dog population, as well as their shorter expected lifespan, but it could result in less accurate estimates of their age trajectory compared to other groups. On the other hand, it is worth noting that the diverse sample sizes of the different breeds and the potential over-representation of popular breeds did not significantly affect the results.

Fourth, our analysis revealed some associations between the various characteristics of the dogs, such as the over-representation of medium-sized dogs among the mixed-breeds or the higher prevalence of brachycephalism among the smallest and largest size groups. Although these relationships could reflect global breeding trends, as well as anatomical or genetical constraints, a strong correspondence could mask or exacerbate the effects of the individual variables.

Another limitation of this study is that to obtain the necessary sample size for the analyses, data were collected from multiple countries. However, we did not account for potential systematic differences among the countries in terms of breed or dog group popularity, and owner rating tendencies, which could introduce a bias to the dataset.

Some limitation also arises from the fact that the data were obtained from volunteer owner reports, namely that the breed, the purebred status, and partly also the body size information also originated from the owners. Although we took steps to control for unreliable size data in purebred dogs, we could only filter out highly unrealistic data for mixed-breeds. Therefore, the latter group may contain wrongly categorized dogs due to incorrect owner guesses or weight issues (such as being strongly under- or overweight).

The expected lifespan estimates of the breeds originated from Finland, while dogs from this country represented less than 0.1% of the total data in our sample. Since the average lifespan of some breeds may vary across countries due to differences in popularity and random breed-stock effects, this could have led to errors in the lifespan categorization. Moreover, due to statistical constrains, we had to create categories for lifespan, which have been done by dividing the sample into three equal-sized groups. However, the thresholds for these groups depend strongly on the sample constitution, and the two extreme groups may not represent objectively short of long life expectancy relative to the middle group.

Finally, we also had to create head-shape categories, even though researchers in this field clearly favor a continuous cephalic index scale [42] due to possible within-breed variation [60], and because there are no generally accepted cut-off values for the categories based on biological or clinical considerations [49]. By creating three equal-sized groups, we aimed to categorize our subjects in the least biased way, however, the threshold values were partly determined by the breeds’ popularity, and thus there is a risk that these groups do not represent the head shape categories well. Moreover, it is also not determined yet which characteristics of the head shape are of importance regarding behavioral and cognitive aging. The cephalic index is calculated from the width and length of the head, so any or both could play a role. In addition, there are alternative ways of quantifying head shape based on different cranial and facial measures (e.g., craniofacial ratio [40]), which could lead to different categorizations of (some) breeds and, consequently, to different results. More in-depth studies are needed to address these issues and identify the candidate factors.

### Conclusions

The present findings provide insight into the impact of morphological and breed-related factors on cognitive and behavioral aging in pet dogs. Our results showed that body size does not exert a gradual effect on behavioral and cognitive aging as it does on lifespan. Quite the contrary, it only affects behavioral and cognitive aging above 30 kg of weight. The earlier onset and slower rate of behavioral and cognitive decline in large-sized dogs is probably a byproduct of their age-related physical decline, which leads to old age behaviors, both directly and via owner care practices, long before their mental decline would begin. However, further targeted experiments and research are needed to confirm this hypothesis and determine the underlying mechanisms.

Nevertheless, our results could help to understand the relationship between lifespan and healthspan, presenting a translational potential for the human ‘longevity dividend’ concept and carrying a practical significance for owners as well. Although giant sized dogs have good mental health until the end, they experience a physical breakdown at an early age, resulting in early death. Toy sized dogs live a long life, but they also carry a much larger risk of age-related mental deterioration. Thus, there is a trade-off between longevity and relative healthspan, however, it seems to concern mostly the two extreme sizes. We found no difference in the aging trajectories among dogs weighing between 6.5–30 kg. Therefore, for those who want a smaller sized dog but do not want to risk severe mental health problems in old age or want a larger sized dog but do not want to risk physical health problems at 7–8 years of age, we recommend a dog from this size range. Based on our results, these dogs have a longer healthspan relative to their expected lifespan than their smaller and larger counterparts. Aside from size, head shape and purebred status were also found to be related to CCD risk, with dolichocephalic breeds and purebreds having generally higher prevalence. These results indicate the need for further research to understand the impact of these factors on dogs' welfare and to develop interventions that can help maintain their physical and mental well-being as they age.

Furthermore, one of the most striking findings from the data was that even though being old is a subjective term without clearly defined distinguishing features from the adult life stage in dogs, the ratio of dogs considered “old” by their owners began to increase in the population uniformly around six years of age, despite more pronounced behavioral changes occurring later in life. This suggests that owners may be aware of age-related changes in their dogs' appearance, health, and behavior, even if the changes are not yet pronounced and underscores the importance of considering the owner's perspective when studying aging in dogs.

### Supplementary Information

Below is the link to the electronic supplementary material.

Supplementary file1 (PDF 173 kb)

Supplementary file2 (XLSX 27 kb)

Supplementary file3 (PDF 309 kb)

Supplementary file4 (XLSX 63 kb)

## Data Availability

The data that support the findings of this study are available on request from the corresponding author, [B.T.].
