# Supplementary material for: Differential behavioral aging trajectories according to body size, expected lifespan, and head shape in dogs
Source: GeroScience. 2023 Sep 23;46(2):1731–54. doi: 10.1007/s11357-023-00945-9 (PMC10828231; doi:10.1007/s11357-023-00945-9)

Supplementary Information 2

Title: When do dogs start to age? Behavioral and cognitive aging are linked to expected lifespan, body size, head shape, and purebred status in dogs

Journal: GeroScience

Authors: Borbála Turcsán*, Enikő Kubinyi
* MTA-ELTE Lendület "Momentum" Companion Animal Research Group, Department of Ethology, Eötvös Loránd University, Budapest, Hungary, borbala.turcsan@gmail.com

Fig S1 Location of the breakpoint in the six raw behavioral characteristics that made up the Liveliness-Trainability factor. The vertical dashed line indicates the location of the breakpoint of the Liveliness-Trainability factor, the bars represent its 95% confidence interval

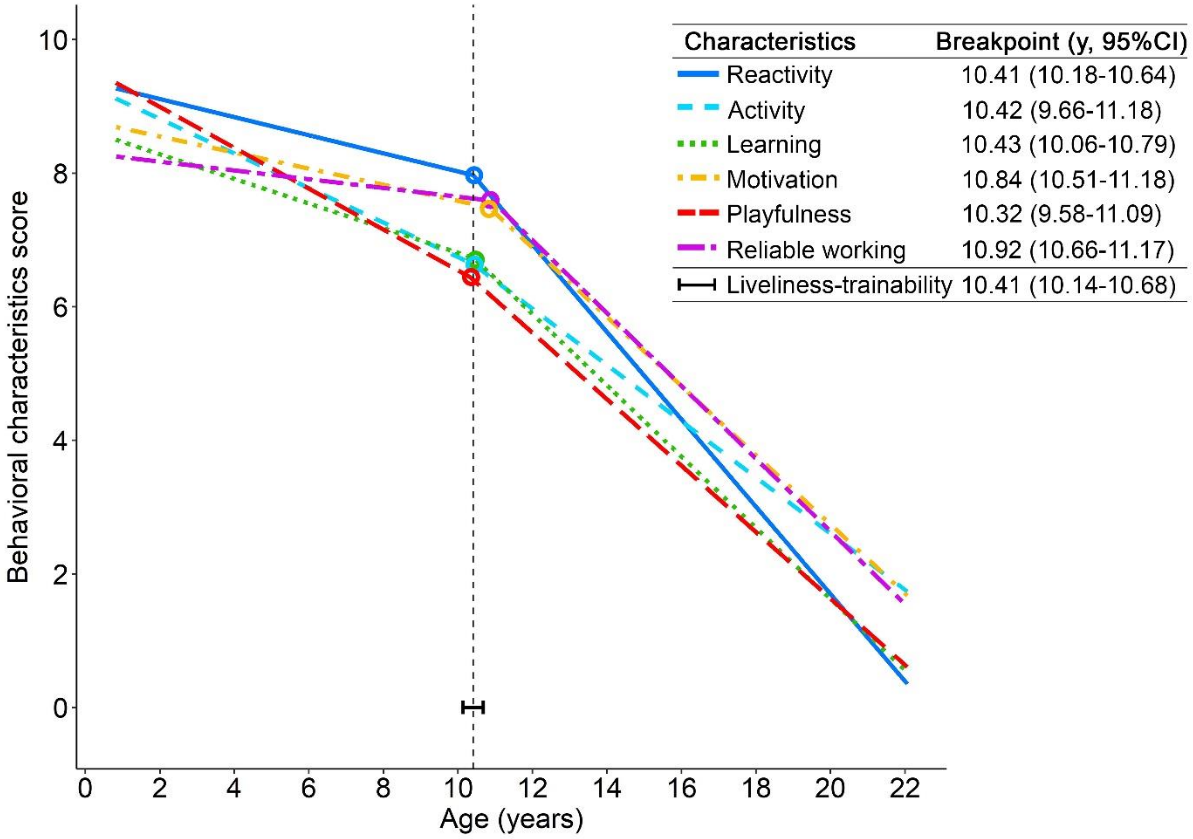

Supplement: Supplementary file 1 — Supplementary file1 (PDF 173 kb) [file 11357_2023_945_MOESM1_ESM.pdf]
