# Supplementary material for: Differential behavioral aging trajectories according to body size, expected lifespan, and head shape in dogs
Source: GeroScience. 2023 Sep 23;46(2):1731–54. doi: 10.1007/s11357-023-00945-9 (PMC10828231; doi:10.1007/s11357-023-00945-9)
Supplement: Supplementary file 3 — Supplementary file3 (PDF 309 kb) [file 11357_2023_945_MOESM3_ESM.pdf]

Supplementary Information 2

Title: Differential behavioral aging trajectories according to body size, expected lifespan, and head shape in dogs

Journal: GeroScience

Authors: Borbála Turcsán*, Enikő Kubinyi

* MTA-ELTE Lendület "Momentum" Companion Animal Research Group, Department of Ethology, Eötvös Loránd University, Budapest, Hungary, borbala.turcsan@gmail.com

Relationship between the grouping variables (Table S1)

Size x lifespan comparison (Chi-square test, N =7,784, χ^2 =4,565.366, $p < 0.001$): Toy dogs were more frequent among the long-lived than among the medium- and short-lived dogs and also more frequent among the medium-lived than among the short-lived dogs. Miniature and medium-small dogs were also more frequent among the long-lived than among the short- and medium-lived dogs, but also more frequent among the short-lived than among the medium-lived dogs. Medium-large dogs, on the other hand, were more frequent among the medium-lived than among the short- and long-lived dogs, and more frequent among the short-lived than among the long-lived dogs. Finally, large dogs were represented only among the short- and medium-lived dogs, while giant dogs were represented only among the short-lived dogs. All pairwise differences are $p < 0.001$.

Size x head shape comparison (Chi-square test, N = 7,241, χ^2 = 2,177.935, $p < 0.001$): Toy, miniature, and giant dogs were more frequent among the brachycephalic than among the meso- and dolichocephalic, toy and miniature were also frequent among the dolichocephalic than among the mesocephalic dogs. Medium-small dogs were more frequent among the dolichocephalic than among the brachy- and mesocephalic dogs, and also more frequent among the brachy- than mesocephalic dogs. Finally, medium-large and large dogs were more frequent among the mesocephalic than among the brachy- and dolichocephalic, and also more frequent among the dolichocephalic than among the brachycephalic dogs. All pairwise differences are $p < 0.001$.

Size x purebred status comparison (Chi-square test, N = 15,174, χ^2 = 925.525, $p < 0.001$): Toy, miniature, large, and giant dogs were more frequent among the purebreds than among the mixed-breeds, while medium-small and medium-large dogs were more frequent among the mixed-breeds. All pairwise differences are $p < 0.001$.

Lifespan x head shape comparison (Chi-square test, N = 6,225, χ^2 =2,516.903, $p < 0.001$): Both long-lived and short-lived dogs were more frequent among the brachycephalic and dolichocephalic than among the mesocephalic dogs, while medium-lived dogs were more frequent among mesocephalic than among dolicho- and brachycephalic dogs. Short- and medium-lived dogs were also more frequent among brachycephalic than among dolichocephalic dogs, and long-lived dogs were more frequent among the dolichocephalic than among the brachycephalic dogs. All pairwise differences are $p < 0.001$.

Table S1 Relationship between the four groupings: body size, lifespan, head shape and purebred status. Size groups were based on Salt et al. (2017); lifespan groups and head shape groups were created by dividing the population with continuous lifespan / cephalic index (CI) information into three ca. equal-sized groups. Percentages in the table are column %

Factor		LIFESPAN			HEAD SHAPE			PUREBRED STATUS		
	Category / group	short-lived	medium-lived	long-lived	brachyceph.	mesoceph.	dolichoceph.	purebred	mixed-breed	TOTAL
SIZE	toy (<6.5 kg)	234 (9.0%)	343 (12.9%)	1066 (41.9%)	679 (27.5%)	22 (0.9%)	308 (13.2%)	1897 (19.1%)	353 (6.8%)	2264 (14.8%)
	mini (6.5 - <9 kg)	225 (8.7%)	29 (1.1%)	799 (31.4%)	448 (18.2%)	70 (2.9%)	172 (7.4%)	1131 (11.4%)	453 (8.7%)	1597 (10.5%)
	medium-small (9- <15 kg)	435 (16.8%)	272 (10.3%)	567 (22.3%)	538 (21.8%)	180 (7.4%)	596 (25.5%)	1552 (15.6%)	1330 (25.5%)	2900 (19.0%)
	medium-large (15- <30 kg)	518 (20.0%)	1274 (48%)	109 (4.3%)	336 (13.6%)	1248 (51.2%)	625 (26.7%)	2693 (27.1%)	2183 (41.8%)	4910 (32.2%)
	large (30-40 kg)	690 (26.7%)	735 (27.7%)	1 (0%)	207 (8.4%)	795 (32.6%)	506 (21.6%)	1763 (17.7%)	661 (12.7%)	2438 (16.0%)
	giant (>40 kg)	487 (18.8%)	0 (0%)	0 (0%)	257 (10.4%)	122 (5.0%)	132 (5.6%)	913 (9.2%)	245 (4.7%)	1161 (7.6%)
LIFESPAN	short-lived (<11.4 yr.)				1168 (57.1%)	205 (9.6%)	1045 (51.1%)	2589 (33.3%)	0 (0%)	2589 (33.3%)
	medium-lived (11.4-12.3 yr.)				412 (20.2%)	1771 (83.0%)	330 (16.1%)	2653 (34.1%)	0 (0%)	2653 (34.1%)
	long-lived (>12.3 yr.)				464 (22.7%)	159 (7.4%)	671 (32.8%)	2542 (33.7%)	0 (0%)	2542 (33.7%)
HEAD SHAPE	brachycephalic (CI>59)							2465 (34.0%)	0 (0%)	2465 (34.0%)
	mesocephalic (51<CI<59)							2437 (33.7%)	0 (0%)	2437 (33.7%)
	dolichocephalic (CI>59)							2339 (32.3%)	0 (0%)	2339 (32.3%)
PUREBRED STATUS	purebred									9949 (59.7%)
	mixed-breed									6727 (40.3%)

Table S2 The oldest year group for each dog group where the sample contained at least ten dogs, which was the minimum number of subjects for the analyses of the CCD-risk prevalence, and the Proportion of "old" dogs variables. The proportion of the dogs with CCD risk and the dogs considered old by their owners in this last year group are also shown

Dog group	Oldest age group with N≥10	CCD-risk prevalence	Proportion of "old" dogs
Lifespan			
Short-lived	16-17 years	47.62%	95. 24%
Medium-lived	17-18 years	52.63%	94.74%
Long-lived	18-19 years	63. 64%	96.00%
Size			
Toy	> 19 years	84.62%	100%
Mini	18-19 years	61.54%	92.31%
Medium-small	17-18 years	57.78%	95.56%
Medium-large	17-18 years	47.06%	94.19%
Large	17-18 years	40.00%	100%
Giant	14-15 years	18.75%	87.50%
Head shape			
Brachycephalic	17-18 years	40.00%	90.00%
Mesocephalic	16-17 years	39.13%	95.65%
Dolichocephalic	18-19 years	76.92%	92.31%
Purebred status			
Purebred	> 19 years	81.82%	100%
Mixed-breed	18-19 years	54.84%	90.32%

Table S3 Relationship between the nine raw behavioral characteristics and the age of the dogs. The results of linear, quadratic, and cubic regressions are shown

Behavioral trait	df2	Regression	R^2	F	P value
Reactivity	16816	Linear	0.246	5478.039	<0.001
	16815	Quadratic	0.286	3363.708	<0.001
	16814	Cubic	0.286	2243.014	<0.001
Activity	16816	Linear	0.251	5623.436	<0.001
	16815	Quadratic	0.254	2863.677	<0.001
	16814	Cubic	0.254	1909.015	<0.001
Learning	16816	Linear	0.222	4811.733	<0.001
	16815	Quadratic	0.242	2682.457	<0.001
	16814	Cubic	0.242	1788.304	<0.001
Motivation	16816	Linear	0.142	2790.899	<0.001
	16815	Quadratic	0.166	1667.501	<0.001
	16814	Cubic	0.166	1116.398	<0.001
Playfulness	16816	Linear	0.261	5935.209	<0.001
	16815	Quadratic	0.264	3016.875	<0.001
	16814	Cubic	0.264	2011.613	<0.001
Working performance	16816	Linear	0.120	2302.113	<0.001
	16815	Quadratic	0.160	1605.751	<0.001
	16814	Cubic	0.161	1076.905	<0.001
Sociality towards owner	16816	Linear	0.044	769.829	<0.001
	16815	Quadratic	0.054	477.681	<0.001

	16814	Cubic	0.056	334.618	<0.001
Sociality towards strangers	16816	Linear	0.005	85.408	<0.001
	16815	Quadratic	0.006	51.570	<0.001
	16814	Cubic	0.010	59.442	<0.001
Sociality towards dogs	16816	Linear	0.037	640.749	<0.001
	16815	Quadratic	0.042	370.849	<0.001
	16814	Cubic	0.045	263.903	<0.001

Table S4 Number of subjects, prevalence of CCD-risk, and proportion of dogs considered “old” by the owner in the nineteen age groups of the full sample (N=16,818)

**Age group**	**N of subjects (% of total sample)**	**CCD-risk prevalence (% in each age group)**	**Proportion of “old” dogs (% in each age group)**
10 months-2 years	816 (4.85%)	1.35%	7.35%
>2-3 years	906 (5.39%)	0.99%	8.28%
>3-4 years	897 (5.33%)	1.11%	10.37%
>4-5 years	1007 (5.99%)	2.38%	13.01%
>5-6 years	1153 (6.86%)	2.17%	18.47%
>6-7 years	1176 (6.99%)	1.96%	24.23%
>7-8 years	1269 (7.55%)	2.99%	36.17%
>8-9 years	1422 (8.46%)	3.59%	49.79%
>9-10 years	1421 (8.45%)	4.93%	65.31%
>10-11 years	1483 (8.82%)	7.82%	74.58%
>11-12 years	1302 (7.74%)	9.60%	81.11%
>12-13 years	1154 (6.86%)	13.43%	89.43%
>13-14 years	1005 (5.98%)	22.39%	94.03%
>14-15 years	724 (4.3%)	36.05%	95.03%
>15-16 years	525 (3.12%)	43.62%	96.76%
>16-17 years	315 (1.87%)	47.62%	95.56%
>17-18 years	158 (0.94%)	57.59%	93.04%
>18-19 years	64 (0.38%)	57.81%	92.19%
>19 years	21 (0.12%)	57.14%	95.24%

Table S5 Parameters of the slopes with 95% of confidence interval (in brackets). The parameters were extracted using the '*slope*' function of the R package *Segmented* (v.1.6-0), separately for each behavioral trait, each dog group, and each segment (before and after the breakpoint)

**Dog group / segment relative to breakpoint**		**Liveliness-Trainability**	**Severity of CCD symptoms**	**CCD-risk prevalence**
Lifespan				
Short-lived	before	**-0.081** (-0.095--0.067)	**0.032** (0.016-0.048)	**0.007** (-0.006-0.02)
	after	**-0.219** (-0.248--0.190)	**0.193** (0.172-0.213)	**0.071** (0.052-0.089)
Medium-lived	before	**-0.063** (-0.078--0.048)	**0.040** (0.029-0.052)	**0.006** (-0.001-0.013)
	after	**-0.213** (-0.235--0.191)	**0.239** (0.214-0.264)	**0.076** (0.064-0.088)
Long-lived	before	**-0.079** (-0.094--0.063)	**0.038** (0.020-0.056)	**0.006** (-0.002-0.014)
	after	**-0.273** (-0.297--0.250)	**0.296** (0.272-0.321)	**0.085** (0.074-0.096)
Body size				
Toy	before	**-0.077** (-0.092--0.062)	**0.044** (0.028-0.060)	**0.009** (-0.002-0.019)
	after	**-0.255** (-0.280--0.230)	**0.304** (0.276-0.331)	**0.090** (0.078-0.102)
Mini	before	**-0.071** (-0.090--0.053)	**0.020** (-0.002-0.042)	**0.004** (-0.005-0.013)

	after	**-0.250** (-0.282--0.218)	**0.242** (0.215-0.268)	**0.077** (0.064-0.089)
Medium-small	before	**-0.074** (-0.086--0.061)	**0.038** (0.025-0.051)	**0.006** (-0.001-0.014)
	after	**-0.236** (-0.260--0.212)	**0.271** (0.246-0.295)	**0.083** (0.070-0.096)
Medium-large	before	**-0.062** (-0.073--0.052)	**0.039** (0.031-0.048)	**0.007** (0.000-0.014)
	after	**-0.231** (-0.248--0.213)	**0.240** (0.220-0.259)	**0.081** (0.064-0.098)
Large	before	**-0.072** (-0.088--0.056)	**0.021** (0.004-0.037)	**0.003** (-0.016-0.022)
	after	**-0.193** (-0.217--0.169)	**0.186** (0.167-0.206)	**0.061** (0.038-0.084)
Giant	before	**-0.074** (-0.108--0.039)	**0.021** (-0.007-0.050)	**0.005** (-0.010-0.020)
	after	**-0.153** (-0.185--0.121)	**0.130** (0.103-0.157)	**0.028** (0.013-0.044)
Head shape				
Brachycephalic	before	**-0.085** (-0.098--0.072)	**0.041** (0.028-0.055)	**0.010** (0.001-0.018)
	after	**-0.217** (-0.250--0.184)	**0.197** (0.172-0.221)	**0.056** (0.043-0.070)
Mesocephalic	before	**-0.072** (-0.085--0.059)	**0.024** (0.008-0.039)	**0.006** (-0.006-0.017)
	after	**-0.258** (-0.289--0.227)	**0.222** (0.200-0.244)	**0.077** (0.052-0.103)
Dolichocephalic	before	**-0.051** (-0.071--0.031)	**0.055** (0.041-0.068)	**0.010** (0.003-0.017)
	after	**-0.215** (-0.233--0.196)	**0.286** (0.257-0.315)	**0.107** (0.093-0.121)
Purebred status				
Purebred	before	**-0.077** (-0.084--0.071)	**0.045** (0.039-0.052)	**0.008** (0.001-0.014)
	after	**-0.244** (-0.257--0.230)	**0.273** (0.259-0.286)	**0.086** (0.079-0.094)
Mixed-breed	before	**-0.073** (-0.082--0.065)	**0.031** (0.024-0.039)	**0.004** (-0.001-0.008)
	after	**-0.224** (-0.239--0.208)	**0.243** (0.228-0.258)	**0.074** (0.067-0.080)
